# Protein Complexes are Central in the Yeast Genetic Landscape

**DOI:** 10.1371/journal.pcbi.1001092

**Published:** 2011-02-24

**Authors:** Magali Michaut, Anastasia Baryshnikova, Michael Costanzo, Chad L. Myers, Brenda J. Andrews, Charles Boone, Gary D. Bader

**Affiliations:** 1 The Donnelly Centre, University of Toronto, Toronto, Ontario, Canada; 2 Banting and Best Department of Medical Research, University of Toronto, Toronto, Ontario, Canada; 3 Department of Molecular Genetics, University of Toronto, Toronto, Ontario, Canada; 4 Department of Computer Science, University of Toronto, Toronto, Ontario, Canada; 5 Department of Computer Science and Engineering, University of Minnesota, Minneapolis, Minnesota, United States of America; University of Zurich, Switzerland

## Abstract

If perturbing two genes together has a stronger or weaker effect than expected, they are said to genetically interact. Genetic interactions are important because they help map gene function, and functionally related genes have similar genetic interaction patterns. Mapping quantitative (positive and negative) genetic interactions on a global scale has recently become possible. This data clearly shows groups of genes connected by predominantly positive or negative interactions, termed monochromatic groups. These groups often correspond to functional modules, like biological processes or complexes, or connections between modules. However it is not yet known how these patterns globally relate to known functional modules. Here we systematically study the monochromatic nature of known biological processes using the largest quantitative genetic interaction data set available, which includes fitness measurements for ∼5.4 million gene pairs in the yeast *Saccharomyces cerevisiae*. We find that only 10% of biological processes, as defined by Gene Ontology annotations, and less than 1% of inter-process connections are monochromatic. Further, we show that protein complexes are responsible for a surprisingly large fraction of these patterns. This suggests that complexes play a central role in shaping the monochromatic landscape of biological processes. Altogether this work shows that both positive and negative monochromatic patterns are found in known biological processes and in their connections and that protein complexes play an important role in these patterns. The monochromatic processes, complexes and connections we find chart a hierarchical and modular map of sensitive and redundant biological systems in the yeast cell that will be useful for gene function prediction and comparison across phenotypes and organisms. Furthermore the analysis methods we develop are applicable to other species for which genetic interactions will progressively become more available.

## Author Summary

Genetic interactions indicate functional dependencies between genes and are a powerful tool to predict gene function. Functionally related genes tend to have similar profiles of genetic interactions. Recently, global scale mapping of quantitative (positive and negative) genetic interactions has been performed. This data clearly shows groups of genes connected by predominantly positive or negative interactions, termed monochromatic groups. These groups often correspond to functional modules, such as biological processes or protein complexes, or connections between modules, but it is not yet known how these patterns globally relate to known functional modules. Here we systematically evaluate the monochromatic nature of known biological processes and their connections in the yeast *Saccharomyces cerevisiae*. We find that 10% of biological processes and less than 1% of inter-process connections are monochromatic. Further, we show that protein complexes are responsible for a surprisingly large fraction of these monochromatic groups. The monochromatic processes, complexes and connections we find chart a hierarchical and modular map of sensitive and redundant biological systems in the yeast cell that will be useful for gene function prediction and comparison across phenotypes and organisms.

## Introduction

One of the major goals in biology is to understand how molecules are organized within the cell, how they interact to mediate biological processes and how process failure leads to disease. Genetic perturbations, such as gene mutations, are often used to better understand the function of a gene and to study the relationship between genotype and phenotype [1]. In budding yeast, most genes (∼80%) are not essential for growth under standard laboratory conditions [1], suggesting that their function is not required under the conditions tested or is compensated by other genes. Exploring mutant phenotypes in the presence of a chemical or an environmental stress [2], along with combining multiple mutations to map genetic interactions [3], [4], [5], have been successful strategies to investigate genetic redundancy. In particular, genetic interactions have proven useful to predict gene function [6] and organize biological processes [7], [8], [9] and are complementary to other functional interaction data such as protein-protein interactions [10]. Here we systematically evaluate how genetic interaction data relates to known biological processes. We next review previous work in this area to place our work into context.

Genetic interactions are observed when the phenotype of a double mutant is unexpected given the phenotypes of both single mutants [11]. With respect to growth, a genetic interaction is classified as either positive (or negative) when the fitness of the double mutant is higher (or lower) than expected. Negative genetic interactions often indicate functional redundancy between two genes, with the extreme case being synthetic lethality (SL) when simultaneous deletion of two otherwise non-essential genes leads to cell death. A biochemical interpretation for this is that the two genes participate in complementary or parallel pathways or complexes [12], [13]. As a result, two complementary pathways tend to be connected by many negative genetic interactions. Positive interactions may indicate a number of biochemical scenarios, but are typically thought of as being within a pathway or complex, such as a linear chain of reactions where the deletion of one gene affects output, but deleting a second gene doesn't further affect output [4].

While the precise relationship of a genetic interaction to its underlying biochemistry is still not completely understood, functionally related genes tend to have similar genetic interaction profiles. Clustering the first large scale genetic interaction maps composed of SL and synthetic sick interactions resulted in clear grouping of genes with correlated genetic interaction profiles [4], [5], [6], [7]. These genes tend to be physically linked as part of the same biochemical pathway, multiprotein complex or physically interacting protein pairs. Genes with correlated profiles tend to encode physically interacting proteins and tend not to interact synthetically with each other [4], [6]. This supports a model where parallel biochemical pathways are connected by a large number of SL interactions [14], [15], [16].

Extending the parallel pathway model, Kelley et al. defined biological modules as clusters of proteins enriched in genetic and physical interactions (“within-module”) connected only by SL genetic interactions (“between-module”) [17]. This approach was further explored by defining modules more generally as a connected graph in a protein interaction network [18] and considering pairs of modules connected by negative interactions [19]. Extending this idea to quantitative positive and negative genetic interactions, Bandyopadhyay et al. and others devised methods to learn protein complexes and their functional relationships [20], [21]. These approaches aim to define functional modules (clusters of functionally related genes, such as pathways or complexes) using large-scale genetic interactions.

By predicting positive and negative interactions on a large scale from metabolic network simulations, Segre et al. discovered that biological modules are often connected by purely positive or negative genetic interactions, and described these as monochromatically pure connections [22]. Quantitative experimentally determined genetic interactions [4], [7] have been interpreted using this concept and found to also contain monochromatic groups of genes, where all genes are connected to each other by predominantly positive or negative genetic interactions. Theoretically, we can consider four monochromatic patterns: monochromatic positive or negative interactions within groups of genes (“within module”) and monochromatic positive or negative connections between groups of genes (“between module”). Only some of these patterns have been related to the underlying biochemical system or systematically explored. Monochromatic positive within module patterns have been shown to correspond to protein complexes or pathways [4], [9], [20], [23], [24]. Monochromatic negative within module patterns have been observed to represent complexes containing essential genes [20], [25]. Also, complexes enriched in genetic interactions tend to be monochromatic positive or negative [25]. Monochromatic positive between module connections have not been related to a biochemical model, but have been observed to occur between functional modules in simulations [22] and between complexes [25]. Monochromatic negative between module connections have been observed in simulations [22] and are expected from the observation that SL interactions (negative) connect parallel pathways [9], [14], [15]. Overall, monochromatic patterns have been linked to various physical modules, however none have been systematically studied in terms of all known pathways and complexes.

Two approaches to systematically study monochromatic patterns are possible. We can either search for monochromatic patterns in the genetic interaction network and interpret the results in terms of known physical and functional modules (such as protein complexes or biological processes), or we can examine all known modules for monochromatic patterns. The former approach has been applied in a non-exhaustive fashion on focused genetic interaction data sets [26], [27], but an exhaustive approach is computationally difficult, given the large size of recently published genetic interaction networks [7], and requires the development of new algorithms. The latter approach uses the knowledge we currently have about functional modules to study monochromatic patterns and this is what we adopt here.

We use current knowledge about biological processes from Gene Ontology annotation [28] combined with the most comprehensive quantitative genetic interaction data set currently available, which includes measurements for 5.4 million gene pairs in normal growth conditions and provides quantitative genetic interaction profiles for ∼75% of all genes in *Saccharomyces cerevisiae* [7]. We first assess the monochromatic nature of biological processes and their connections and find that only 10% of biological processes and less than 1% of inter-process connections are monochromatic. We next explore various features that may explain these monochromatic patterns and show that protein complexes are responsible for a surprisingly large fraction of them. Significantly more genetic interactions than expected are attributed to complexes and genes encoding protein complex members have more genetic interactions and are essential more often than expected. This work shows the importance of protein complexes in contributing to monochromatic patterns in quantitative genetic interaction networks and generates a hierarchical and modular map of sensitive and redundant biological systems in the yeast cell.

## Results

### ∼10% of biological processes are monochromatic

To study the monochromatic nature of known biological processes, we used the most recent data set of quantitative genetic interactions, generated by Synthetic Genetic Array (SGA) analysis [7]. Known processes were defined by the Gene Ontology (GO) biological process (BP) classification system as annotated to yeast genes by the Saccharomyces Genome Database (SGD). GO annotations in yeast are the most complete for any organism and there is no other comparable database of biological processes for yeast. Processes can represent canonical pathways, like fatty acid biosynthesis, and also more general processes, like DNA repair. Each process is described by a standard name and a set of genes annotated to it. We considered all GO processes in yeast where member genes were connected by at least one SGA interaction (∼1000 processes).

**Figure 1:**
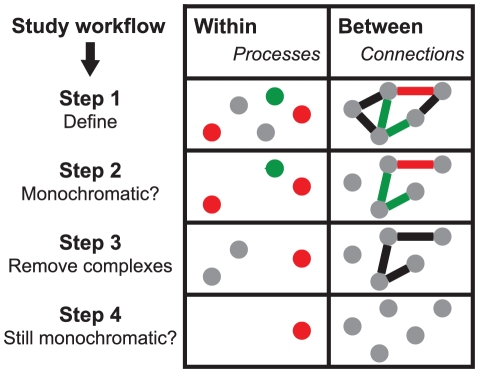
Monochromatic analysis overview. We evaluate the monochromatic nature of biological processes (Within) and of the connections between processes (Between). Circles represent processes and links between them represent the set of SGA genetic interactions that connect genes within the processes. The color represents the monochromatic nature of the processes and connections (green is monochromatic positive, red is monochromatic negative, grey is non-monochromatic). We first define the processes and their connections and then evaluate their monochromatic nature. In the third step we remove various features, such as genes whose products are part of a complex and finally analyze the resulting change in monochromatic nature of the processes and connections.

We defined the monochromatic score as the relative ratio of positive to negative interactions occurring within a given process (set of genes). To assess how likely these scores are to occur by chance, we computed Z-scores using randomization that maintains the network topology (permutation of the gene names). We can then identify unexpected monochromatic patterns by their high Z-scores (Figure 1). Highly positive Z-scores characterize monochromatic positive processes and highly negative Z-scores characterize monochromatic negative processes (Methods).

Not all gene pairs are tested in the SGA data set, thus processes have variable genetic interaction coverage. Because monochromatic patterns are more confident for processes that have an increased coverage of genetic interactions, we selected high coverage processes based on the number of corresponding genes present and connected in the SGA genetic interaction network (Methods). For a given coverage level, we computed the ratio of monochromatic processes among all covered processes. We found that this ratio ranges from 7 to 9% (Table 1). Thus, just under 10% of SGA covered biological processes are monochromatic.

**Table 1:** Monochromatic processes at various SGA coverage levels.

Coverage	Covered processes	Monochromatic processes	Ratio (%)
0	1031	68	6.6
0.2	1019	68	6.7
0.4	833	66	7.9
0.6	566	50	8.8
0.8	99	9	9.0
1	25	2	8.0

For each coverage cutoff, this table indicates the number of processes covered and how many of them are monochromatic with the ratio it represents.

Choosing a coverage cut-off of 0.6 that reasonably traded higher coverage for a larger number of terms, we identified 50 monochromatic processes, including 5 positive and 45 negative (Dataset S1). Thus, even though positive interactions are often presumed to be acting within processes [4], [9], there are actually more processes that are monochromatic negative.

**Figure 2:**
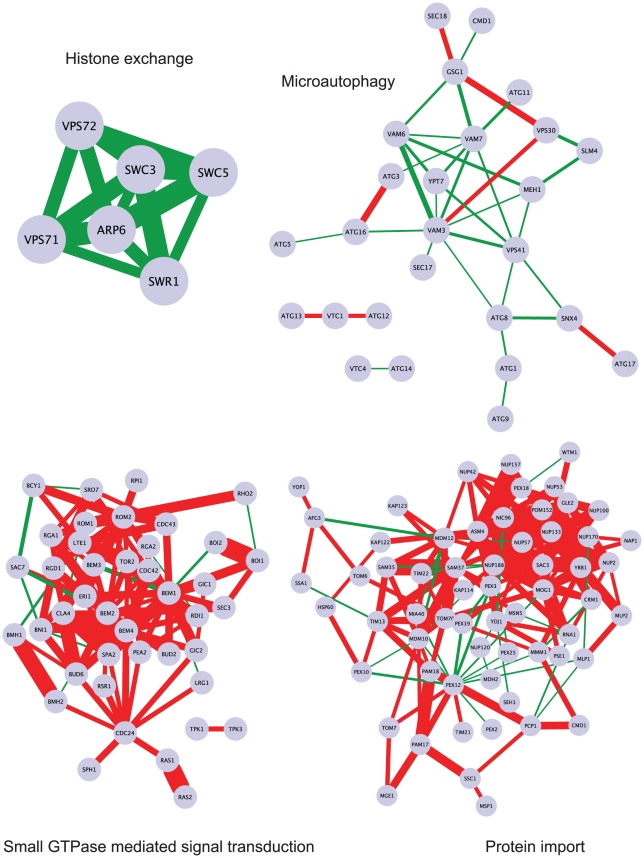
Examples of monochromatic biological processes. Circles represent genes and are labeled by the common gene name. Edges represent SGA genetic interactions between the genes. The color indicates the sign of the SGA score (green is positive, red is negative) and the width of the edge is proportional to the absolute value of the SGA score (epsilon) (the thicker the edge, the stronger the interaction). Biological processes are represented as four different networks labeled by the process name: ‘microautophagy’ and ‘histone exchange’ are monochromatic green, whereas ‘protein import’ and ‘small GTPase mediated signal transduction’ are monochromatic red. The networks were produced using Cytoscape [43].

**Figure 3:**
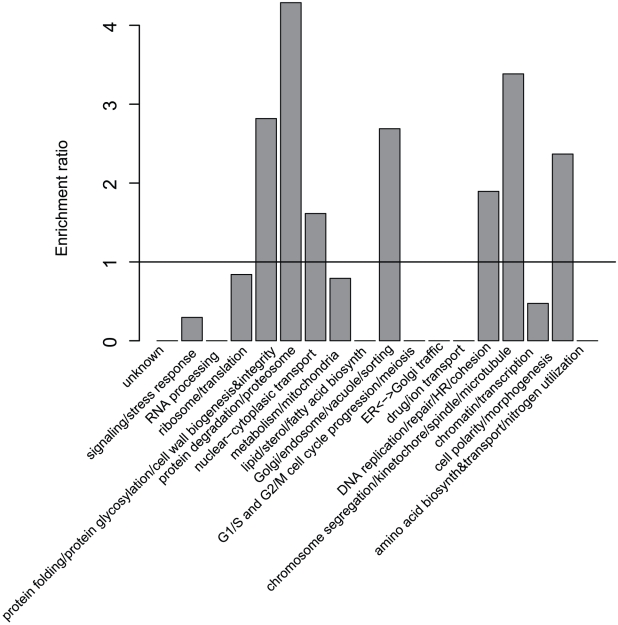
Monochromatic processes are enriched in specific high-level functional categories. The count of processes in each category is normalized by the background distribution for all processes. For example, 35 processes among all 2,501 yeast processes are in the category ‘protein degradation/proteasome’ in the background distribution whereas 3 among the 50 monochromatic processes are in that category, resulting in an enrichment ratio of 3/50 * 2501/35 = 4.3.

Monochromatic processes are functionally diverse, but also biased. For instance, microautophagy and histone exchange are monochromatic positive whereas protein import and small GTPase mediated signal transduction are monochromatic negative (Figure 2). Globally, monochromatic processes are enriched in specific functions, including chromosome segregation/microtubule and protein degradation/proteasome (Figure 3). Further, positive monochromatic processes are generally much smaller (< = 40 genes) than negative ones (∼100 genes) and are more specific in the GO hierarchy (Figure S1).

### Monochromatic connections between biological processes are rare

To investigate the monochromatic nature of connections between biological processes, we defined an inter-process connection as the set of genetic interactions linking genes annotated to two processes, excluding those annotated to both. The monochromatic nature of an inter-process connection was assessed by measuring enrichment for positive and/or negative interactions relative to the background distribution in the entire genetic interaction network (Methods). The resulting p-value was used as a score to select the most monochromatic connections.

The coverage of a connection was assessed by the number of genetic interactions tested in SGA compared to all possible interactions between the two processes. Using a range of coverage cut-offs, we found that only ∼0.27% of covered connections are monochromatic (Table 2). For instance, the pair of processes ‘glycosylation’ (49 genes) and ‘tRNA modification’ (51 genes) is connected by 44 positive genetic interactions and 29 negative interactions while we expect 20 positive and 36 negative interactions by chance. This connection is thus highly enriched in positive interactions. The process ‘tRNA modification’ is connected to ‘cell wall organization’ (191 genes) by 80 positive genetic interactions and 296 negative interactions while we expect 76 positive and 137 negative interactions by chance. This predominantly negative connection indicates that many genes from both processes buffer each other.

**Table 2:** Monochromatic connections at various SGA coverage levels.

Coverage	Covered connections	Monochromatic connections	Ratio (%)
0	525727	1394	0.27
0.2	525710	1394	0.27
0.4	525380	1394	0.27
0.6	511671	1386	0.27
0.8	240680	609	0.25

For each coverage cutoff, this table indicates the number of connections covered and how many of them are monochromatic with the ratio it represents.

With a reasonable coverage cut-off of 0.6, we identified 1,387 monochromatic connections, including 614 positive (44%) and 773 negative connections (56%) (Dataset S2). Previous analyses suggested that positive interactions often occur between genes acting together in the same pathway or complex [4], [9] and negative genetic interactions tend to occur between genes implicated in redundant processes [9], thus we expect connections to be monochromatic negative. However, we observe an even distribution of negative to positive connections showing that between process connections are not predominantly negative. In addition, our results show that connections between GO-defined processes are rarely monochromatic.

### Protein complexes explain most monochromatic processes

**Figure 4:**
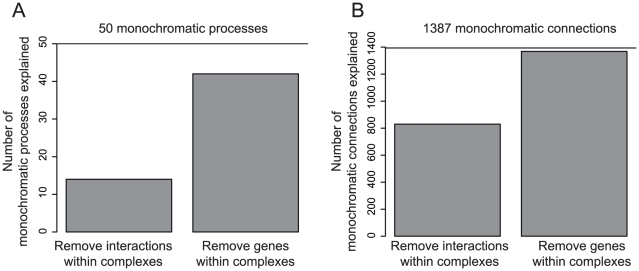
Protein complexes explain most monochromatic processes and connections. A) Some monochromatic processes are no longer monochromatic when we remove the interactions occurring within complexes. Most monochromatic processes are no longer monochromatic when we remove the genes encoding proteins in complex. B) Half of the monochromatic connections are no longer monochromatic when we remove the interactions occurring within complexes. Most monochromatic connections are no longer monochromatic when we remove the genes encoding proteins in complex.

We noticed that the monochromatic processes identified above often contain protein complexes or parts of complexes. In fact, all monochromatic processes but six contain at least one gene encoding a member of a complex. Since complexes enriched in genetic interactions tend to be monochromatic [25], we evaluated the contribution of all protein complexes to the monochromatic patterns we observed. To do this, we removed genes or interactions corresponding to protein complexes from the SGA genetic interaction data set and repeated our monochromatic analysis described above. When we removed all genes encoding proteins that are part of a complex, most (82%) of the monochromatic processes identified above were no longer monochromatic (Figure 4). Interestingly, a few new processes became monochromatic after this, but these were either small processes with positive interactions or very large processes with negative interactions (Figure S1). When we removed interactions occurring within complexes, a smaller number (28%) of the monochromatic processes were explained. These results hold for various coverage cut-offs (Table 1 in Text S1). As a control, we showed that removing the same number of random genes encoding proteins not in any complex did not have the same effect (Kolmogorov-Smirnov (KS) test p<4 10^−4^, Figure S2). We confirmed our results using another curated set of yeast protein complexes that combines predictions from high-throughput and literature data to form a consensus set [29] (called *Consensus*). This data set includes existing complexes defined previously by Pu et al. and Hart et al. [30], [31]. Again most monochromatic processes (90%) were explained by genes encoding proteins in complexes (Figure S3) and this result holds for various coverage cut-offs (Table 3 in Text S1). This indicates that genes whose products are part of a complex are the main contributors to the monochromatic genetic interaction patterns we see in GO biological processes.

We also considered three other features that may contribute to our monochromatic patterns: essential genes, low single mutant fitness genes and duplicate genes. Essential genes (tested as hypomorphic or conditional alleles in genetic screens) are known to have many negative interactions [7], genes which have a strong effect on yeast fitness, as measured by growth rate when deleted (i.e. a low single mutant fitness) similarly tend to show many negative interactions [7], and duplicate genes often buffer each other and thus are typically connected by a strong negative interaction [32]. We removed each of these gene sets in turn and evaluated the effect on our observed monochromatic patterns. All these features partly explain the monochromatic patterns previously identified but not as much as the genes encoding proteins in complex (Figure S3). In addition, these features are highly overlapping with the set of genes encoding proteins in complexes (Figure S4). For example, 60% of essential genes are in a complex. Thus, we presume that the effect of these features on monochromatic processes is minor and mainly due to their correlation with protein complexes.

### Protein complexes explain most monochromatic connections

To measure to what extent the features presented above explain the monochromatic nature of connections, we adopted the same strategy of removing each feature in turn and analyzing the resulting change in number of monochromatic connections. Again, genes encoding proteins in complexes explained most monochromatic connections (98%) whereas the other features only partly explained the monochromatic connections (Figure 4, Figure S3). This result holds for various coverage cut-offs (Table 2 in Text S1) and was confirmed using *Consensus*, the alternative set of protein complexes (Figure S3, Table 4 in Text S1). Removing the same number of random genes not in a complex did not have the same effect on the monochromatic pattern (KS p = 0, Figure S2). An example of a monochromatic connection explained is the positive connection between the ‘mitotic sister chromatid cohesion’ and the ‘regulation of glucose metabolic process’ processes (Figure S5). This monochromatic positive connection is mainly due to positive genetic interactions between genes encoding proteins from the ‘GID complex’ and the ‘AMP-activated protein kinase complex’ on one side, and the ‘replication fork protection complex’ and the ‘Ctf18 RFC-like complex’ on the other side. When we removed the genes encoding proteins in a complex, these processes were no longer connected by a monochromatic positive connection. These results suggest that genes encoding proteins in a complex play a key role in the monochromatic connections between yeast GO biological processes.

### 30% more SGA interactions than expected are attributed to complexes

Since protein complexes are important in explaining monochromatic GO processes in the genetic interaction network, we examined their contribution at the genetic interaction level. We defined a genetic interaction as involving a complex if at least one gene in the interaction encodes a protein that is part of a complex. It is expected that 49% (93,383) of all observed SGA interactions (189,996) involve a protein complex gene since 49% (2,801,630) of all tested gene pairs involve a protein complex gene. Surprisingly, we found that 63% (119,871) of the observed SGA genetic interactions involve complexes, or 28% more than expected. This significant bias (Fisher p<10^−5^) is present globally and for both negative and positive interactions (Table 3). Since some genes might be noisy and cause a false signal by virtue of having an extreme number of interactions, we repeated the analysis with progressively more stringent sets of genetic interactions, defined by the SGA score [25]. At each increased stringency level, we found the result to be stronger and more significant (Table 3 in Text S1). In addition, the global degree distribution confirms that genes encoding proteins in complexes are more likely to have more interacting partners than genes encoding proteins not in any complex (KS p<2 10^−4^). Finally, to check the robustness of our results to our definition of complexes, we confirmed them using the *Consensus* data set used above [29] (Methods, Table 10 in Text S1). These results indicate that genes encoding proteins in complexes are more likely to genetically interact than genes encoding proteins not in any complex.

**Table 3:** Number of interactions involving complexes.

Obs/Exp	Complex (CI)	Non complex (NCI)	P-value
All	1.28	0.73	<10^−16^
Positive	1.31	0.70	<10^−16^
Negative	1.27	0.74	<10^−16^

For all (positive, negative) interactions, the p-value is computed by a Fisher's Exact test between the expected and observed number of interactions involving complexes (CI) or not (NCI).

As previously noted, most monochromatic negative complexes contain essential genes [20], [25]. More generally, we found that essential genes are highly enriched within complexes, 225% more than expected (Fisher p = 0)(Table 4 in Text S1) and this result also holds when considering only genes present in the genetic interaction network (Fisher p<10^−60^, Table 5 in Text S1). Furthermore, the number of essential genes per complex has a broad distribution (Figure S6): many complexes are composed of all essential genes, and a high proportion (57%) of protein complexes are essential (contain at least one essential gene). This bias was also present in the *Consensus* data set (Tables 6–9 in Text S1). This result may be related to the increased number of genetic interactions involving protein complexes observed above, as essential genes are known to have more genetic interactions than non-essential genes [7]. This observation may be influenced by experimental preference for studying essential genes, but this can’t fully explain the results, as many high-throughput methods have been used to defined yeast complexes [29]. Altogether, the above results show that protein complexes play an important role in the monochromatic genetic landscape of biological processes and more generally in yeast growth.

## Discussion

Monochromatic patterns have been used to identify biological processes and other functional modules [22], [26], [27]. In this work, we ask to which extent known processes show these monochromatic patterns. To answer this question, we systematically studied the monochromatic landscape in yeast using known biological processes as defined by GO annotation and a large network of genetic interactions. We found that approximately 10% of GO-defined biological processes that are sufficiently covered by genetic interactions are monochromatic and less than 1% of all pairs of processes interact monochromatically. We observe that monochromatic processes tend to be predominantly negative whereas between process connections are evenly distributed between positive and negative.

Interestingly, we found that protein complexes explain most of the monochromatic signal present in GO processes and are disproportionately important for yeast growth (are involved in more genetic interactions and contain more essential genes compared to non-complex genes). We hypothesize that protein complexes are more sensitive to perturbation and more difficult to buffer, either because it is more difficult to duplicate the functionality of an entire complex or that complexes participate in more processes compared to individual proteins (Figure S7). Previous work observed that protein complexes are often monochromatic [7], [20] but we show for the first time that the monochromatic patterns identified within and between biological processes are mainly driven by protein complexes.

We chose GO as the representation of known biological processes since it is the most comprehensive resource available. KEGG and SGD YeastCyc also make available pathway information, but these are limited mostly to metabolic pathways and do not cover as many genes as GO, making a general analysis difficult. In addition, GO organizes processes hierarchically, which clarifies the relationships between processes and sub-processes. However, this makes processes highly overlapping. The number of monochromatic processes depends on this overlap. To assess the effect of overlap, we applied our method on the reduced ontology GO Slim, which contains fewer and less overlapping terms compared to the full GO. We identified 11 monochromatic processes among 26 covered processes (Dataset S3, Dataset S4). Similarly to the full GO analysis, most (73%) of the monochromatic processes are no longer monochromatic when removing genes encoding proteins complex members (Figure S8). In addition we applied the analysis to the MIPS Comprehensive Yeast Genome Database (CYGD) Functional Category (FunCat), an alternative classification of biological processes [33]. This classification is hierarchical but, unlike GO, every category has only one root category. Since genes can be annotated to several categories, there is some overlap between categories, but to a lesser extent compared to GO. We found that 8% of the covered processes were monochromatic (similar to GO)(Dataset S5, Dataset S6) and protein complexes explained most (78%) of them (Figure S8). This confirms that protein complexes play an important role in the monochromatic nature of biological processes, even when considering less overlapping process definitions.

While we used the most comprehensive data for genetic interactions and process annotations available, much data is still missing which can impact our results. Some processes may appear monochromatic because not all interactions are known, or some processes may not be considered because they are lacking interactions. To account for the lack of completeness of the genetic interaction network, we only considered highly covered processes. The results are presented for a reasonable level of coverage and confirmed with multiple coverage thresholds. Also, monochromatic processes likely exist that contain genes that are not covered by our best efforts to collect the most comprehensive annotation available (GO, FunCat, KEGG, YeastCyc). As annotation improves, we expect the monochromatic map to expand. Also, complementary unsupervised approaches, such as clustering or motif detection, can be used to find monochromatic patterns that we miss. It will be interesting to see how many monochromatic modules found by these methods are not currently captured in GO.

The monochromatic processes, complexes and connections we find chart a hierarchical and modular map of sensitive and redundant biological systems in the yeast cell (Dataset S1, Dataset S2, Figure S9). Our results indicate that the genetic interaction network is enriched in interactions involving protein complexes, monochromatic connections between processes are rare and protein complexes play an important role in defining monochromatic patterns within and between processes. These results are illustrated on the example map presented in Figure S9.

Our map holds for genetic interactions measured in standard laboratory growth conditions. It will be interesting to compare it with other maps constructed based on genetic interactions defined using phenotypic readouts other than yeast growth or not in standard laboratory conditions [34], [35] and from other species for which genetic interactions will progressively become more available, such as *Caenorhabditis elegans* [36], [37], *Drosophila melanogaster* [38] or mammalian cells.

## Methods

### Genetic interaction network

The genetic interaction data are from the most recent and comprehensive study in yeast obtained by the Synthetic Genetic Array technique (SGA) in normal growth conditions [7]. This data set consists of 191,890 pair-wise interactions between 4,415 genes derived from 1,712 full genome screens. Each interaction is characterized by the epsilon score, a quantitative genetic interaction measure, and a p-value, indicating confidence. This score can be positive or negative, indicating a positive or a negative interaction. When different measurements are available for a single gene (i.e. from several screened alleles of essential genes), we merge all interactions (this occurs for 35 genes). If two screens give opposite scores, we remove both. If two screens give scores of the same sign, we keep the one with the best p-value. The resulting network contains 166,401 pair-wise interactions among 4,415 genes.

### Biological processes

We downloaded the annotation of the yeast genome provided by SGD [39] on September 7th, 2009. For the Biological Process ontology, all genes annotated to one specific GO term are up-propagated to all parents of that GO term. We don't consider non-manually reviewed annotations (IEA evidence code). We only consider GO terms with more than one observed interaction between its genes and with less than 200 genes in the genetic interaction matrix, otherwise the random networks are not different enough to assess the statistical significance of the monochromatic scores. We are left with a set of 1,031 processes in yeast with genetic interactions in SGA. In addition, we downloaded the functional categories from FunCat [33] and filtered out those not referring to biological processes (16: protein with binding function or cofactor requirement; 70: subcellular localization; 73: cell type localization; 75: tissue localization; 77: organ localization, 18: regulation of metabolism and protein function; 98: classification not yet clear-cut; 99: unclassified proteins).

### Assessing the coverage of a biological process (*i.e.* a GO term)

For a given GO term, its genes can be present in the genetic interaction network or not. If present, they contribute to the monochromatic nature only if they are connected by an SGA interaction within the GO term. We assess the coverage of the GO term by the minimum value of the two following ratios: (i) the number of genes in the GO term and in the SGA genetic interaction network over the total number of genes in the GO term; (ii) the number of connected genes in the GO term over the total number of genes in the GO term and in the genetic interaction network.

### Assessing the monochromatic level of biological process (*e.g.* a GO term)

We define the monochromatic score of a GO term as the relative ratio of positive to negative interactions observed between the genes in that GO term (equation 1).
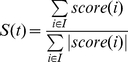
(1)where I is the set of interactions occurring between two genes from the GO term t. This score ranges from −1, meaning fully monochromatic negative, to +1, meaning fully monochromatic positive.

We then generate random networks by shuffling the labels of the original genetic interaction network, which preserves the network topology. For each GO term, we compute a series of monochromatic scores obtained with the random genetic interaction networks and use this distribution of scores to compute a Z-score (equation 2).
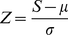
(2)where S is the monochromatic score to be standardized, μ is the mean of the random scores and σ the standard deviation of the random scores. GO terms with a Z-score larger than 1.6 in absolute value are selected as monochromatic.

### Assessing the monochromatic level of a connection between two GO terms

A connection between two GO terms is formed by all interactions between one gene belonging to one GO term and another gene belonging to the other GO term. Genes belonging to both GO terms were not considered. The coverage is computed as the ratio of the number of tested pairs over the number of possible pairs. For a given number of tested interactions, we computed the expected number of positive and negative interactions, following the global ratio of observed interactions in the full network. We considered the number of observed positive and negative interactions and tested if these numbers significantly differed from those expected using Fisher's Exact Test. We then selected the most monochromatic connections with p<0.01.

### High-level functional categories

To group processes into high-level functional categories depicted in Figure 3, we used the yeast gene annotations provided in [7] where 4,414 genes are associated to 18 functional categories. Each biological process is then associated to the functional category that most of its genes are annotated to. To analyze the set of monochromatic processes, we counted the number of processes annotated in each of the high-level functional categories. We then computed the enrichment compared to the background distribution of all processes. The multi-functionality of a gene used in Figure S7 was assessed by the number of processes this gene is involved in, which was computed as the number of GO biological process annotations for each gene restricted to the functionally distinct set of GO terms described in [40].

### Protein complexes

We used the cellular component part of the Gene Ontology to define protein complexes in yeast. We considered all the children of the GO term macromolecular complex (GO:0032991). Each term defined a protein complex formed by the genes directly annotated to that term (not considering IEA annotations). This way, we defined 347 complexes encoded by 1,795 genes (Dataset S7). We used this data set in the analysis, unless otherwise stated. We also considered a recent curated consensus of protein complexes in yeast [29]. This *Consensus* set is a combination of predictions from high-throughput data and curated literature data and consists of 409 complexes.

### Feature selection

We used two ways to remove the effect of complexes: i) remove all genes that encode proteins that are part of at least one complex; ii) remove the interactions that occur between two genes encoding proteins from the same complex, but leaving the genes in place (in the former case all interactions involving these genes were removed whereas in the latter case only interactions between two genes encoding proteins of the same complex were removed). Thus when attempting to explain monochromatic patterns, we considered the following five features, removing either genes or interactions:

- Essential genes: genes described as essential for normal yeast growth by the Saccharomyces Genome Deletion Project [41] (http://www-sequence.stanford.edu/group/yeast_deletion_project/downloads.html)
- Low SMF genes: genes with low single mutant fitness [7] (10% lowest)
- Complex genes: genes part of at least one complex (see the definition of the complexes above)
- Complex interactions: interactions occurring between two genes part of at least one complex (see the definition of the complexes above)
- Intra-duplicate gene interactions: interactions occurring between two duplicate genes. The set of duplicate pairs is a combination whole genome duplication (WGD) [42] and smaller-scale duplicates (SSD) [32]. SSD are defined based on sequence similarity with an alignment that covers more than 50% of the length of the longer protein and a BLAST e-value<10^−10^.

The overlap between the above features was computed using the Jaccard coefficient.

### Interaction bias

To examine the role of protein complexes at the interaction level, we studied all possible gene pairs. A given pair was considered as involved in a complex if at least one of the genes encodes a protein that is part of a complex. In other words, we partitioned the genes into two classes: 1) complex genes (CG): genes that encode a protein that is part of at least one complex and 2) non-complex genes (NCG): genes that encode a protein that is not part of any complex. We partitioned the interactions into two classes: 1) complex interactions (CI): interactions involving at least one gene of the class CG and 2) non-complex interactions (NCI): interactions occurring between two genes of the class NCG. Assuming that the complexes do not significantly affect the structure of the genetic interaction network, we expect the distribution of interaction number among the classes to be the same as the background distribution of all tested pairs. For each interaction class (CI/NCI) we computed the ratio of number of observed/expected interactions.

### Network visualization

The networks were produced using Cytoscape [43]. The position of the monochromatic processes in the GO tree is available as a Cytoscape file in Dataset S8.

## Supporting Information

Dataset S1 The file contains the list of the monochromatic processes (described by GO identifier and name), number of genes associated with each process, number of genes in the SGA data, number of genes connected by SGA interactions, number of observed/positive/negative interactions, monochromatic score/Z-score and coverage ratios.
(0.03 MB XLS)

Dataset S2 The file contains the list of monochromatic connections described by GO identifiers and names of both processes, number of genes associated to both processes, number of genes in common between the two processes, numbers of tested/positive/negative interactions, coverage of the possible interactions, numbers of expected positive/negative interactions and monochromatic score.
(0.30 MB XLS)

Dataset S3 The file contains the list of the monochromatic processes restricted to GO slim processes (described by GO identifier and name), number of genes associated to this process, number of genes in the SGA data, number of genes connected by SGA interactions, number of observed/positive/negative interactions, monochromatic score/Z-score and coverage ratios.
(0.02 MB XLS)

Dataset S4 The file contains the list of monochromatic connections restricted to GO slim processes (described by GO identifiers and names) of both processes, number of genes associated to both processes, number of genes in common between the two processes, numbers of tested/positive/negative interactions, coverage of the possible interactions, numbers of expected positive/negative interactions and monochromatic score.
(0.03 MB XLS)

Dataset S5 The file contains the list of the monochromatic processes defined by FunCat (described by identifier and name), number of genes associated to this process, number of genes in the SGA data, number of genes connected by SGA interactions, number of observed/positive/negative interactions, monochromatic score/Z-score and coverage ratios.
(0.02 MB XLS)

Dataset S6 The file contains the list of monochromatic connections between processes defined by FunCat, number of genes associated to them, number of genes in common between them, numbers of tested/positive/negative interactions, coverage of the possible interactions, numbers of expected positive/negative interactions and monochromatic score.
(0.09 MB XLS)

Dataset S7 The file describes the complexes used in the study (extracted from Component Cellular of Gene Ontology) and the number of essential genes they contain.
(0.06 MB XLS)

Dataset S8 The file enables to visualize the map of monochromatic processes using the Cytoscape software.
(0.03 MB ZIP)

Figure S1 A) This network represents the monochromatic processes and their parents in the GO hierarchy. Nodes are processes (GO terms) and edges are parent/child relationships oriented from child to parent. The size of the nodes represents the size of the processes (number of genes associated with the process and present in the genetic interaction network). The color of the node represents the status of the process: monochromatic positive (green), monochromatic negative (red), not monochromatic (grey). The network is provided in Cytoscape format as a supplementary file. The next two plots show the distributions of the process sizes for all/monochromatic positive/monochromatic negative processes for B) the normal situation and C) when we remove genes encoding proteins in complex.
(1.72 MB EPS)

Figure S2 Remove random genes and assess monochromatic nature of A) processes or B) connections. The effect is due to complexes and not to the fact that we remove a large set of genes.
(2.26 MB EPS)

Figure S3 The barplots show how many A) monochromatic processes or B) monochromatic connections are explained by various features (explained means they are no longer monochromatic when removing the given feature).
(0.99 MB EPS)

Figure S4 Overlap between the monochromatic related features in terms of genes, computed using the Jaccard coefficient.
(0.86 MB EPS)

Figure S5 A node represents a gene (and the protein it encodes) and green (or red) edges represent positive (or negative) genetic interactions (the thicker the edge, the higher the interaction score). Proteins in the same complex are grouped together in a purple oval labeled by the name of the complex. At a higher level, proteins which are in the same process are grouped together in a grey rectangle labeled by the name of the process in bold.
(2.21 MB EPS)

Figure S6 The number of genes compared to the number of essential genes in each protein complex and the distribution of the % of essential genes in complexes for A) all genes and B) genes present in the genetic interaction network.
(1.25 MB EPS)

Figure S7 The multifunctionality of a gene is assessed by the number of processes this gene is involved in. The distributions of these multifunctionality indexes show that genes encoding proteins in complex are more likely to be multifunctional than genes encoding proteins not in any complex.
(0.93 MB EPS)

Figure S8 Application of our method to less overlapping biological processes from GO Slim and FunCat. We removed random genes and assessed the monochromatic nature of A) processes and B) connections. The barplots show how many A) monochromatic processes or B) monochromatic connections are explained by various features (explained means they are no longer monochromatic when removing the given feature).
(0.45 MB EPS)

Figure S9 This schema illustrates the monochromatic map for genetic interactions in yeast emphasizing the importance of complexes and the distribution of monochromatic patterns within and between processes. Blue circles represent genes and the proteins they encode, purple ovals represent protein complexes and grey boxes represent biological processes constituted by proteins and complexes. Lines represent SGA genetic interactions between genes (green is positive, red is negative). When the gene encodes a protein in a complex, the line end is a square connected to the complex. The schema indicates that many genetic interactions occur within protein complexes (e.g. 1), but also between genes encoding proteins in complexes and other genes (e.g. 2). Genetic interactions between two genes encoding proteins not in any complex are much less common (e.g. 3). Complexes play an important role in defining monochromatic patterns within and between processes. Monochromatic connections between processes are rare (e.g. 4).
(2.88 MB EPS)

Text S1 This supplementary information file contains various tables for additional analyzes.
(0.12 MB PDF)
