# Supplementary material for: Protein Complexes are Central in the Yeast Genetic Landscape
Source: PLoS Comput Biol. 2011 Feb 24;7(2):e1001092. doi: 10.1371/journal.pcbi.1001092 (PMC3044758; doi:10.1371/journal.pcbi.1001092)
Supplement: Text S1 — This supplementary information file contains various tables for additional analyzes. (0.12 MB PDF) [file pcbi.1001092.s018.pdf]

Table of Contents

Table 1. Monochromatic processes (GO). **1**
Table 2. Monochromatic connections (GO). **1**
Table 3. Monochromatic processes (*Consensus*). **2**
Table 4. Monochromatic connections (*Consensus*). **2**
Table 5. Interaction bias for various SGA scores (*GO*). **2**
Table 6. Essential and non-essential genes (GO). **2**
Table 7. Essential and non-essential genes screened (GO). **3**
Table 8. Essential and non-essential genes (*Consensus*). **3**
Table 9. Essential and non-essential genes screened (*Consensus*). **3**
Table 10. Interaction bias for various SGA scores (*Consensus*). **3**

Coverage	#Monochromatic	Remove genes in complex	Remove interactions within complex
0	68	51 (75%)	27 (40%)
0.2	68	51 (75%)	27 (40%)
0.4	66	49 (74%)	25 (38%)
0.6	50	42 (84%)	14 (28%)
0.8	9	8 (89%)	4 (44%)
1	2	2 (100%)	2 (100%)

Table 1. Monochromatic processes (GO).
Number of monochromatic processes which are no longer monochromatic when removing either genes or interactions in complexes.

Coverage	#Monochromatic	Remove genes in complex	Remove interactions within complex
0	1394	1368 (98%)	830 (60%)
0.2	1394	1368 (98%)	830 (60%)
0.4	1394	1368 (98%)	830 (60%)
0.6	1387	1361 (98%)	829 (60%)
0.8	614	614 (100%)	435 (71%)

Table 2. Monochromatic connections (GO).
Number of monochromatic connections which are no longer monochromatic when removing either genes or interactions in complexes.

Coverage	#Monochromatic	Remove genes in complex	Remove interactions within complex
0	68	50 (74%)	15 (22%)
0.2	68	50 (74%)	15 (22%)
0.4	66	50 (76%)	14 (21%)
0.6	50	45 (90%)	7 (24%)
0.8	9	9 (100%)	2 (22%)
1	2	2 (100%)	0 (0%)

Table 3. Monochromatic processes (*Consensus*).
Number of monochromatic processes which are no longer monochromatic when removing either genes or interactions in complexes from the *Consensus* set of complexes.

Coverage	#Monochromatic	Remove genes in complex	Remove interactions within complex
0	1394	1394 (98%)	697 (50%)
0.2	1394	1394 (98%)	697 (50%)
0.4	1394	1394 (98%)	697 (50%)
0.6	1387	1387 (98%)	691 (50%)
0.8	614	611 (99%)	327 (53%)

Table 4. Monochromatic connections (*Consensus*).
Number of monochromatic connections which are no longer monochromatic when removing either genes or interactions in complexes from the *Consensus* set of complexes.

Score Cut	Pval Cut	All	Cx expected	Cx obs	Ratio	Pval
0.08	0.05	189786	93280	119754	1.28	<1E-16
0.1	0.05	126195	62025	81448	1.31	<1E-16
0.12	0.05	87125	42822	56960	1.33	<1E-16
0.14	0.05	61996	30471	40917	1.34	<1E-16
0.16	0.05	45175	22204	30104	1.36	<1E-16
0.08	0.01	129980	63885	83317	1.30	<1E-16
0.1	0.01	91980	45208	60511	1.34	<1E-16
0.12	0.01	67584	33218	45086	1.36	<1E-16
0.14	0.01	51080	25106	34356	1.37	<1E-16
0.16	0.01	39575	19451	26796	1.38	<1E-16

Table 5. Interaction bias for various SGA scores (*GO*).
For different cutoffs on the SGA score (Score Cut) and p-value (Pval Cut), we examine the partition of all observed interactions (All) between interactions involving complexes (Cx obs) and not. The ratio compares this observed number to what we expect (Cx expected). Pval is the result of a Fisher test.

Fisher p<2.E-16	In complex	Not in complex
Essential genes	684	438
Non essential genes	1111	4378

Table 6. Essential and non-essential genes (GO).

Comparison of the number of genes encoding proteins in GO complexes or not in complex for essential genes versus non-essential genes.

Fisher p<2.E-16	In complex	Not in complex
Essential genes	194	91
Non essential genes	828	3302

Table 7. Essential and non-essential genes screened (GO).
Comparison of the number of genes encoding proteins in GO complexes or not in complex for essential genes versus non-essential genes only considering genes present in the genetic interaction network.

Fisher p<2.E-16	In complex	Not in complex
Essential genes	599	523
Non essential genes	1029	4460

Table 8. Essential and non-essential genes (*Consensus*).
Comparison of the number of genes encoding proteins in *Consensus* complexes or not in complex for essential genes versus non-essential genes.

Fisher p<2.E-16	In complex	Not in complex
Essential genes	164	121
Non essential genes	782	3293

Table 9. Essential and non-essential genes screened (*Consensus*).
Comparison of the number of genes encoding proteins in *Consensus* complexes or not in complex for essential genes versus non-essential genes only considering genes present in the genetic interaction network.

Score Cut	Pval Cut	All	Cx expected	Cx obs	Ratio	Pval
0.08	0.05	189786	84652	110765	1.31	<1E-16
0.1	0.05	126195	56288	75402	1.34	<1E-16
0.12	0.05	87125	38861	52638	1.35	<1E-16
0.14	0.05	61996	27653	37648	1.36	<1E-16
0.16	0.05	45175	20150	27654	1.37	<1E-16
0.08	0.01	129980	57976	77153	1.33	<1E-16
0.1	0.01	91980	41027	56125	1.37	<1E-16
0.12	0.01	67584	30145	41820	1.39	<1E-16
0.14	0.01	51080	22784	31813	1.4	<1E-16
0.16	0.01	39575	17652	24750	1.4	<1E-16

Table 10. Interaction bias for various SGA scores (*Consensus*).
For different cutoffs on the SGA score (Score Cut) and p-value (Pval Cut), we examine the partition of all observed interactions (All) between interactions involving complexes (Cx obs) and not. The ratio compares this observed number to what we expect (Cx expected). Pval is the result of a Fisher test.
